# Hypoxia-induced inflammation: Profiling the first 24-hour posthypoxic plasma and central nervous system changes

**DOI:** 10.1371/journal.pone.0246681

**Published:** 2021-03-04

**Authors:** Louise A. Mesentier-Louro, Barbara Rangel, Laurel Stell, M. Ali Shariati, Roopa Dalal, Abinaya Nathan, Ke Yuan, Vinicio de Jesus Perez, Yaping Joyce Liao

**Affiliations:** 1 Department of Ophthalmology, Stanford University, School of Medicine, Stanford, California, United States of America; 2 Department of Biomedical Data Science, Stanford University, School of Medicine, Stanford, California, United States of America; 3 Department of Pulmonary Medicine, Stanford University, School of Medicine, Stanford, California, United States of America; 4 Divisions of Pulmonary Medicine, Boston Children’s Hospital, Boston, Massachusetts, United States of America; 5 Department of Neurology, Stanford University, School of Medicine, Stanford, California, United States of America; University of Florida, UNITED STATES

## Abstract

Central nervous system and visual dysfunction is an unfortunate consequence of systemic hypoxia in the setting of cardiopulmonary disease, including infection with SARS-CoV-2, high-altitude cerebral edema and retinopathy and other conditions. Hypoxia-induced inflammatory signaling may lead to retinal inflammation, gliosis and visual disturbances. We investigated the consequences of systemic hypoxia using serial retinal optical coherence tomography and by assessing the earliest changes within 24h after hypoxia by measuring a proteomics panel of 39 cytokines, chemokines and growth factors in the plasma and retina, as well as using retinal histology. We induced severe systemic hypoxia in adult C57BL/6 mice using a hypoxia chamber (10% O_2_) for 1 week and rapidly assessed measurements within 1h compared with 18h after hypoxia. Optical coherence tomography revealed retinal tissue edema at 18h after hypoxia. Hierarchical clustering of plasma and retinal immune molecules revealed obvious segregation of the 1h posthypoxia group away from that of controls. One hour after hypoxia, there were 10 significantly increased molecules in plasma and 4 in retina. Interleukin-1β and vascular endothelial growth factor were increased in both tissues. Concomitantly, there was significantly increased aquaporin-4, decreased Kir4.1, and increased gliosis in retinal histology. In summary, the immediate posthypoxic period is characterized by molecular changes consistent with systemic and retinal inflammation and retinal glial changes important in water transport, leading to tissue edema. This posthypoxic inflammation rapidly improves within 24h, consistent with the typically mild and transient visual disturbance in hypoxia, such as in high-altitude retinopathy. Given hypoxia increases risk of vision loss, more studies in at-risk patients, such as plasma immune profiling and in vivo retinal imaging, are needed in order to identify novel diagnostic or prognostic biomarkers of visual impairment in systemic hypoxia.

## Introduction

Systemic hypoxia is a common cause of central nervous system (CNS) dysfunction in many diseases, such as pulmonary hypertension, congestive heart failure [1], cardiac arrest [2, 3], high altitude disease [4, 5], obstructive sleep apnea [6, 7], drowning [8, 9], and most recently SARS-CoV-2 infection [10]. The CNS is particularly vulnerable to hypoxia because the brain [11] and retina [12] consume high levels of oxygen. In humans exposed to high-altitude hypoxia, it is common to experience visual disturbances, such as changes in color vision [13–15], high altitude retinopathy [16, 17], optic disc edema [18, 19] and alterations in multiple electroretinography (ERG) parameters [20]. Rarely, high-altitude hypoxia can lead to irreversible vision loss due to nonarteritic anterior ischemic optic neuropathy [21]. Fundus photography of high altitude retinopathy and optic neuropathy revealed prominent retinal vascular changes including retinal hemorrhages [17, 22], vascular engorgement and tortuosity and disc hyperemia [23, 24], consistent with a combination of hypoxia-induced ischemia and inflammation [25]. CNS effects of systemic hypoxia outside the eye include headache and other symptoms of acute mountain sickness (nausea, dizziness, fatigue) and high altitude cerebral edema, which is a life-threatening stage [5, 26], memory disturbance and depression [7].

Consistent with symptoms of visual disturbance, electrophysiologic measurements at high altitude or hypobaric hypoxia have shown retinal changes suggesting altered function of the inner and outer retina [20, 27]. The retinal ganglion cells in the inner retina seem to be particularly susceptible to transient hypoxia, as changes in the N95 component of the ERG (generated by those cells) occur as soon as 5 min after inhalation of 12% O_2_ by healthy adults (20.9% O_2_ at sea level) [28]. Unfortunately, electrophysiology is a complex technique to perform in experimental settings and often uncomfortable for patients. However, advancement of noninvasive ophthalmic imaging means techniques such as optical coherence tomography (OCT) can be rapidly deployed to assess changes in the human eye as a result of hypoxia. Human OCT studies showed increased thickness of the retinal nerve fiber layer and ganglion cell layer after ascent to high altitude [21, 29]. OCT is fast, non-invasive and easy to perform in humans and animals, making it extremely useful to monitor changes in the retina, including at shorter exposures to hypoxia [30].

In animal models, we have previously described that 48h systemic hypoxia caused limited cell loss in the outer retina and no neuronal loss in the inner retina, but induced prominent optic nerve glia response, endoplasmic reticulum stress and loss of oligodendrocytes [30], which can lead to axonal dysfunction and visual disturbance due to impaired saltatory signal transmission. In the cerebral cortex, hypobaric hypoxia leads to a progressive increase in the levels of hypoxia-inducible factor 1-*a*, vascular endothelial growth factor (VEGF) and Angiopoietin-2, all of which plateau or decrease after the first week in hypoxia [31], suggesting that important molecular signaling occur in the CNS within one week of hypoxia. Hypoxia effects in the CNS tissue are exacerbated by the release of pro-inflammatory mediators by glial cells [32, 33], and systemic inflammation in the setting of hypobaric hypoxia leads to cerebral edema facilitated by the interaction between astrocytes and microglia through toll-like receptors, upregulation of Aquaporin-4 (AQP4) and water permeability [34, 35]. In the mouse retina, we have shown that 3-week systemic hypoxia leads to retinal angiogenesis [36], which develops between 2 and 3 weeks in hypoxia [37] and is consistent with increased vascular density in brain striatum, hippocampus, cerebellum and medulla oblongata after 2 weeks of hypoxia [38].

Given the accessibility of the retina and optic nerve as part of the CNS and the ease of ophthalmic imaging using OCT to identify ophthalmic biomarkers of systemic hypoxia, we induced severe systemic hypoxia for 1 week in adult mice and examined the posthypoxic OCT changes at 2 time points within 24h. To assess posthypoxic inflammation, we profiled retinal and plasma inflammatory and other molecular changes using a Luminex 39-immune molecule assay. Finally, we analyzed retinal and optic nerve histologic changes and compared them with that of OCT and immune profiling, with focus on glial cells in the retina and optic nerve, since we found that glial cells were most impacted after 48h hypoxia [30].

## Results

### Retinal edema during posthypoxic recovery

**Fig 1:**
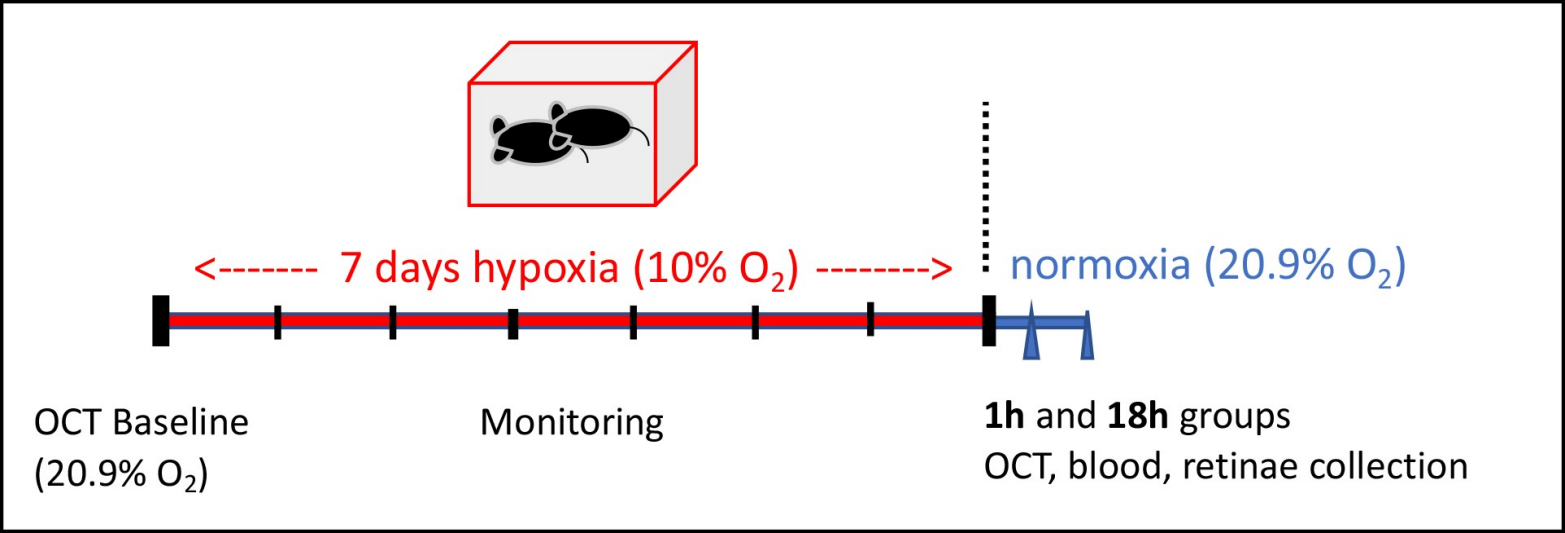
Experimental design. We housed adult mice in a hypoxia chamber (10% oxygen) [30, 93] for one-week and measured changes in optical coherence tomography (OCT), plasma immune profiling using 39-molecule Luminex immune assay, and immunohistochemistry at two time points: 1h and 18h after hypoxia. These 2 time points represented the median time of blood and retinae collection, and the 1h time point represents the fastest possible time point after hypoxia. Assessment during hypoxia was not possible because animals would have to be removed from the hypoxic chamber. Naïve animals housed at room air (20.9% O_2_) were used as control.

**Fig 2:**
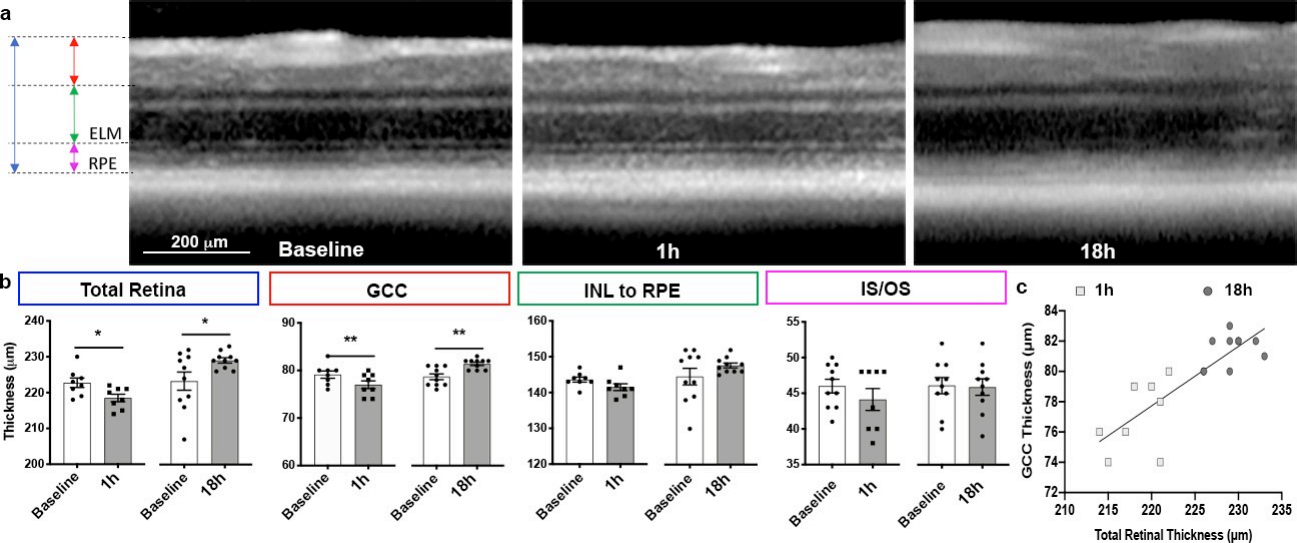
Post hypoxic retina structural changes. (a) High magnification of circle scans acquired using optical coherence tomography in vivo imaging at baseline, 1h and 18h posthypoxia. Colored arrows on the left indicate how retina was segmented; arrow colors in a match label colors in b. (b) Bar graph of retinal thickness at baseline and after hypoxia. For each region of the retina, side-by-side graphs show results for 1h and 18h groups. (c) XY plot of TRT versus GCC for hyperacute and acute groups shows a separate clustering according to their retina thickness. TRT: total retinal thickness, GCC: ganglion cell complex, INL: inner nuclear layer, RPE: retinal pigmented epithelium, IS/OS: inner and outer segments of photoreceptors.

We induced severe systemic hypoxia by exposing adult C57BL/6 mice to one week of 10% O_2_ (Fig 1) and used optical coherence tomography (OCT) to assess structural changes of posthypoxic retinae. Curiously, 1h after hypoxia, there was a significant 5 μm decrease in total retina thickness (TRT) on OCT compared to baseline. Layer-by-layer segmentation showed that significant thinning was in the inner retina in the ganglion cell complex (GCC) but not outer retinal layers (Fig 2 and Table 1). In contrast, after 18h, there was dramatic thickening of the TRT (10.6 μm) relative to the 1h group (P = 0.0002) and 5.8 μm relative to baseline (P = 0.0320). Again, the OCT changes were only significant in the inner retina in the GCC by 4.4 μm (P <0.0001) but not the outer retina. There was significant correlation between TRT and GCC (r = 0.8375, P <0.0001). There were no changes in the other layers in the 1h and 18h groups compared with baseline. Thinning of the GCC on OCT may reflect selective vulnerability of the inner retina in hypoxia, and then peripapillary edema developed, likely as a result of posthypoxic inflammation.

**Table 1:** Optical coherence tomography measurements at baseline and at 1h and 18h posthypoxia.

	**Baseline**			**1h**					
Segmentation	Mean	SEM	n	Mean	SEM	n	P-value paired test	Δ (18h-1h)	%change
TRT	222.8	1.3	8	218.5	1.1	8	0.013	10.6	4.9
GCC	79.13	0.8	8	77	0.8	8	0.004	4.4	5.7
INL to RPE	143.6	0.7	8	141.5	1	8	0.093	6.2	4.4
IS/OS	46	1	10	44.1	1.5	8	0.234	1.8	4.1
	**Baseline**			**18h**					
Segmentation	Mean	SEM	n	Mean	SEM	n	P-value paired test	P-value Tukey*	
TRT	223.3	2.6	10	229.1	0.7	10	0.032	0.0002	
GCC	78.8	0.6	10	81.4	0.3	10	0.001	<0.0001	
INL to RPE	144.6	2.3	10	147.7	0.7	10	0.199	0.1545	
IS/OS	46.1	1.1	10	45.9	1.2	10	0.913	0.5596	

TRT: total retinal thickness, GCC: ganglion cell complex, INL to RPE: inner nuclear layer to retinal pigmented epithelium, IS/OS: photoreceptors inner segments and outer segments, SEM: standard error of the mean.

*Comparison between hyperacute and acute groups, different eyes.

### Increase in 10 inflammatory proteins in the plasma 1h after hypoxia

**Fig 3:**
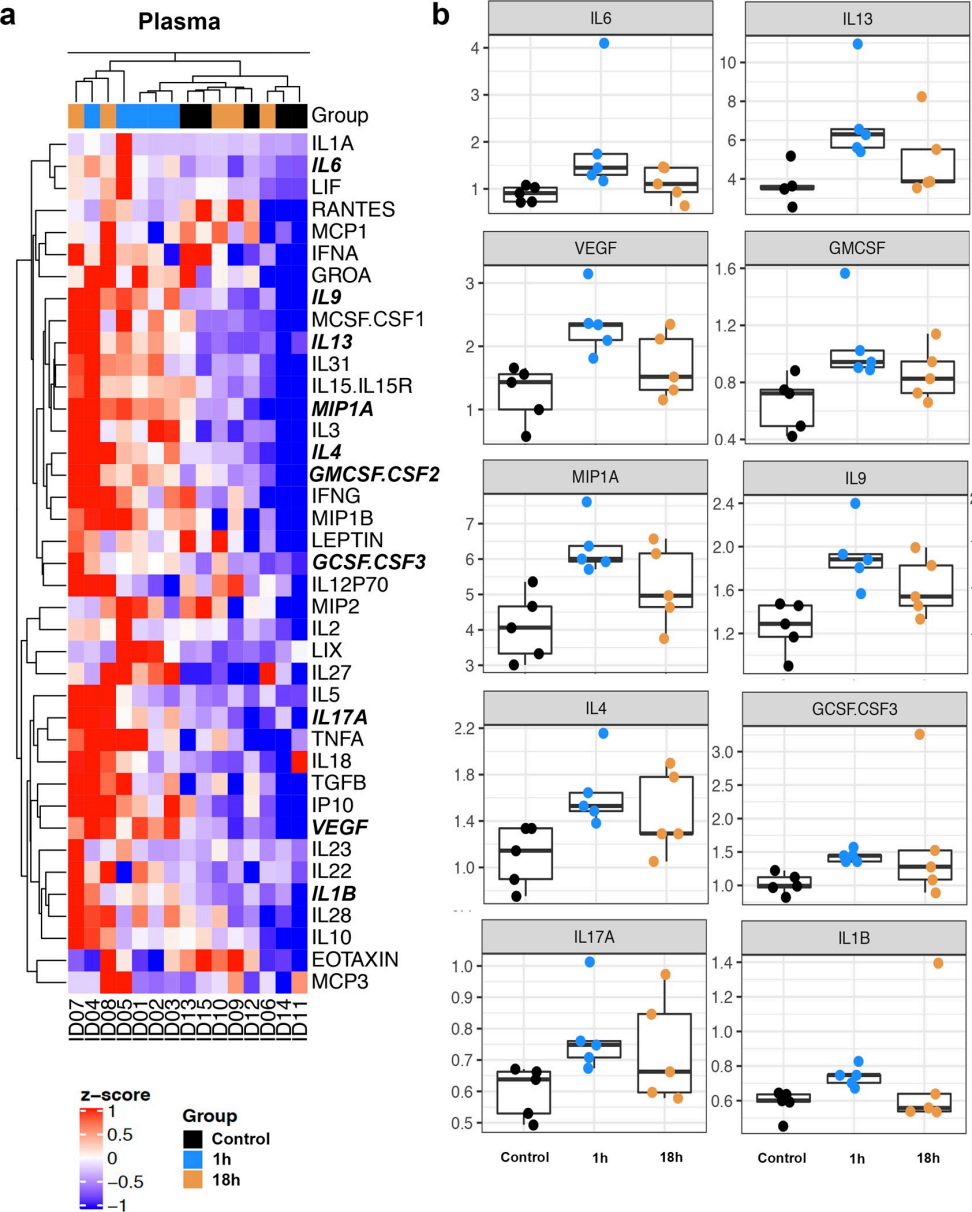
Hierarchical clustering of 39 immune molecules (a) and box plots (b) of the 10 most significantly increased molecules (P = 0.008) in the plasma 1h posthypoxia. Vertical dendrogram denotes 39 molecules, and horizontal dendrogram denotes mouse plasma by ID number. The 10 significantly changed molecules in (b) are bolded and italicized in (a). *Abbreviations*: IL1A: interleukin-1α, IL6: interleukin-6, LIF: leukemia inhibitory factor, RANTES (Regulated on Activation, Normal T Cell Expressed and Secreted), MCP1: monocyte chemoattractant protein-1, IFNA: interferon alpha-A, GROA: Growth-regulated alpha, IL9: interleukin-9, MCSF.CSF1: macrophage colony stimulating factor or colony-stimulating factor 1, IL13: interleukin-13, IL31: interleukin-31, IL15.IL15R: interleukin-15 and interleukin-15 receptor, MIP1A: macrophage inflammatory protein 1-α, IL3: interleukin-3, IL4: interleukin-4, GMCSF.CSF2: granulocyte-macrophage colony stimulating factor or colony-stimulating factor 2, IFNG: interferon γ, MIP1B: macrophage inflammatory protein 1-β, GCSF.CSF3: granulocyte colony-stimulating factor or colony stimulating factor 3, IL12P70: interleukin-12, MIP2: macrophage inflammatory protein 2, IL2: interleukin-2, IL27: interleukin-27, IL5: interleukin-5, IL17A: interleukin-17A, TNFA: tumor necrosis factor-alpha, IL18: interleukin-18, TGFB: transforming growth factor beta, IP10: interferon γ-induced protein 10, VEGF: vascular endothelial growth factor, IL23: interleukin-23, IL22: interleukin-22, IL1B: interleukin-1β IL28: interleukin-28, IL10: interleukin-10, MCP3: monocyte chemotactic protein-3.

To determine whether hypoxia induced systemic inflammation, we asked whether there was alteration of plasma 39 immune molecules 1h after exposure to hypoxia. Hierarchical clustering shows that all samples collected at 1h segregated away from that of controls and there was obvious overlap between the control group and the samples collected 18h after exposure to hypoxia (Fig 3A). In the 1h group, 10 molecules were significantly increased by up to 2 folds compared with controls using permutation test (Table 2 and Fig 3B). Using Mann-Whitney test, there were 7 additional molecules that were significantly different between the 1h and control groups (S1 Fig and S1 Table). No molecules in the 18h group were significantly changed compared to controls. Of the 10 molecules that were significantly increased in the 1h group (Fig 3B), the 5 most increased were: interleukin- (IL) 6, IL-13, vascular endothelial growth factor (VEGF), granulocyte-macrophage colony-stimulating factor (GM-CSF), and macrophage inflammatory protein 1-alpha (MIP1α).

**Table 2:** Molecular changes one hour after exposure to 1w hypoxia.

Plasma 1h		
*Protein*	*Ratio to control*	*P-value permutations*
IL6	*2*.*192*	0.008
IL13	*1*.*898*	0.008
VEGF	*1*.*891*	0.008
GMCSF.CSF2	*1*.*628*	0.008
MIP1A	*1*.*549*	0.008
IL9	*1*.*526*	0.008
IL4	*1*.*499*	0.008
GCSF.CSF3	*1*.*399*	0.008
IL17A	*1*.*303*	0.008
IL1B	*1*.*263*	0.008
Retina 1h		
*Protein*	*Ratio to control*	*P-value permutations*
VEGF	*2*.*107*	0.008
IL1B	*1*.*898*	0.008
IL22	*1*.*456*	0.008
MCP3	*1*.*244*	0.008

There were 10 immune molecules that were significantly increased in the plasma and 4 molecules that were significantly increased in the retina (P = 0.008, permutations test). *Abbreviations*: IL6: interleukin-6, IL13: interleukin-13, VEGF: vascular endothelial growth factor, GMCSF.CSF2: granulocyte-macrophage colony-stimulating factor or colony-stimulating factor 2, MIP1A: macrophage inflammatory protein 1-alpha, IL9: interleukin-9, IL4: interleukin-4, GCSF.CSF3: granulocyte colony-stimulating factor or colony-stimulating factor 3, IL17A: interleukin-17A, IL1B: interleukin-1-beta, IL22: interleukin-22, MCP3: monocyte chemotactic protein-3.

### Increase in 4 immune proteins in the retina 1h after hypoxia

**Fig 4:**
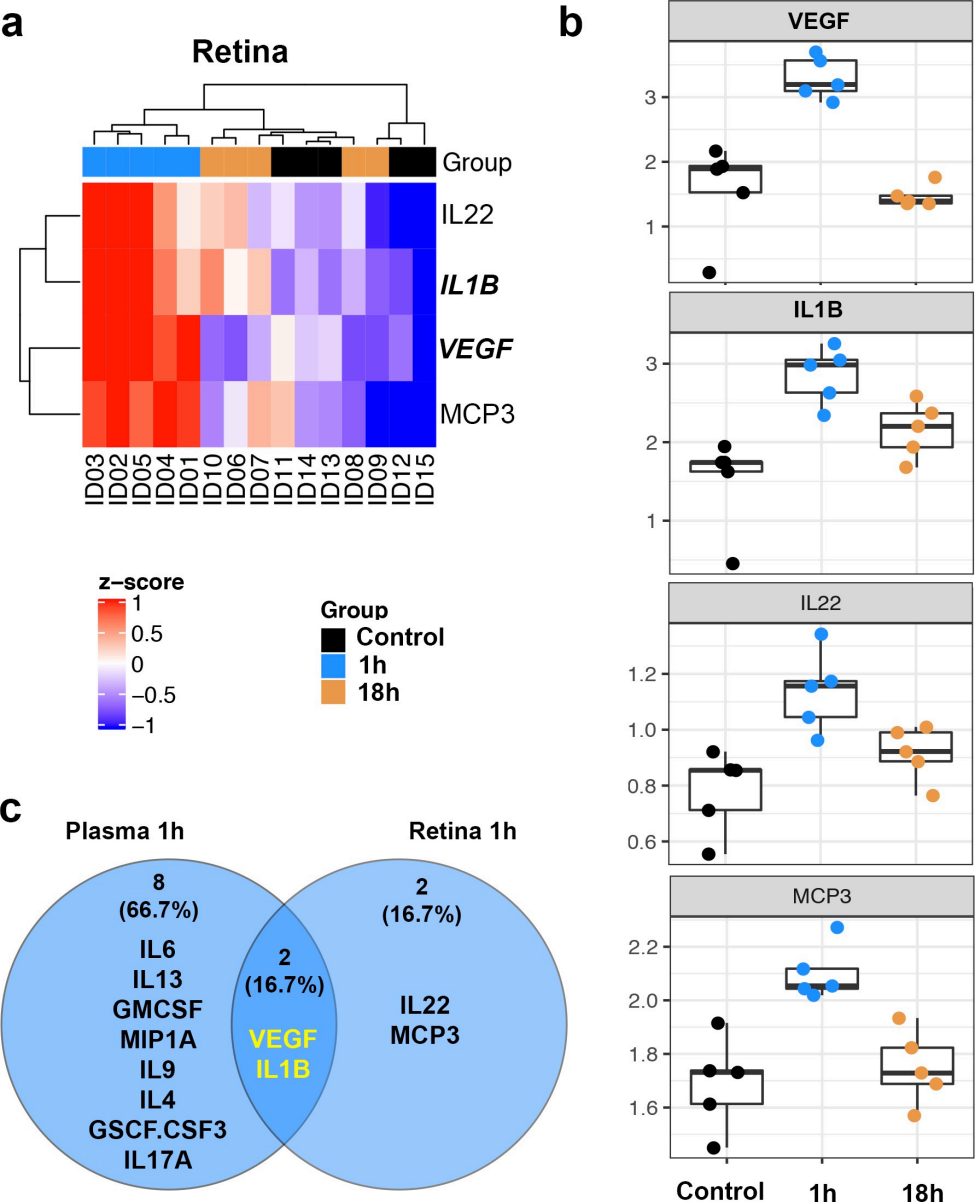
Increased inflammatory molecules in the retina 1h and 18h posthypoxia. (a) Dendogram showing the hierarchical clustering of 4 significantly increase proteins in the retina of 1h (blue), 18h (orange) and control (black) groups. Samples of the 1h group are clustered on the left side of the dendogram, and samples of the 18h group are mixed with control samples on the right side. (b) Box plots of the 4 molecules that were significantly increased (P = 0.008) in the 1h retina and then returned to control levels in the 18h group. Each sample is identified by an ID number, and the samples with same ID across retina and plasma (see Fig 1) are from the same donor. (c) Venn diagram showing how significantly changed molecules are compared between plasma and retina. VEGF and IL1B are the only molecules that are significantly changed in both groups, and they are both upregulated. IL22: interleukin-22, IL1B: interleukin-1β, VEGF: vascular endothelial growth factor, MCP3: monocyte chemotactic protein-3.

To determine whether plasma changes reflected inflammatory changes in the CNS, we performed the same immune profiling in the retinae (S2 Fig) from the same animals as above. We analyzed retina because retinal changes in systemic hypoxia are well documented and it is arguably the most accessible part of the CNS. One hour after hypoxia, 4 inflammatory molecules were significantly increased in the retina. After 18h, no molecules were changed compared with controls. Hierarchical clustering of these 4 proteins shows obvious clustering of the 1h group eyes away from the controls and the 18h group (Fig 4A). Comparing retina and plasma, VEGF (2.1 x increased) and IL-1β (1.9x) were increased in the plasma (Table 2), while IL-22 (1.5x) and monocyte-chemotactic protein 3 (MCP3) (1.2x) were not (Fig 4A–4C and Table 2). Overall, we found nice similar pattern in both retina and plasma immune profiling; there was increased inflammatory molecules 1h after hypoxia but this normalized after 18h.

### Retinal glial changes in water and ion transport during posthypoxic recovery

**Fig 5:**
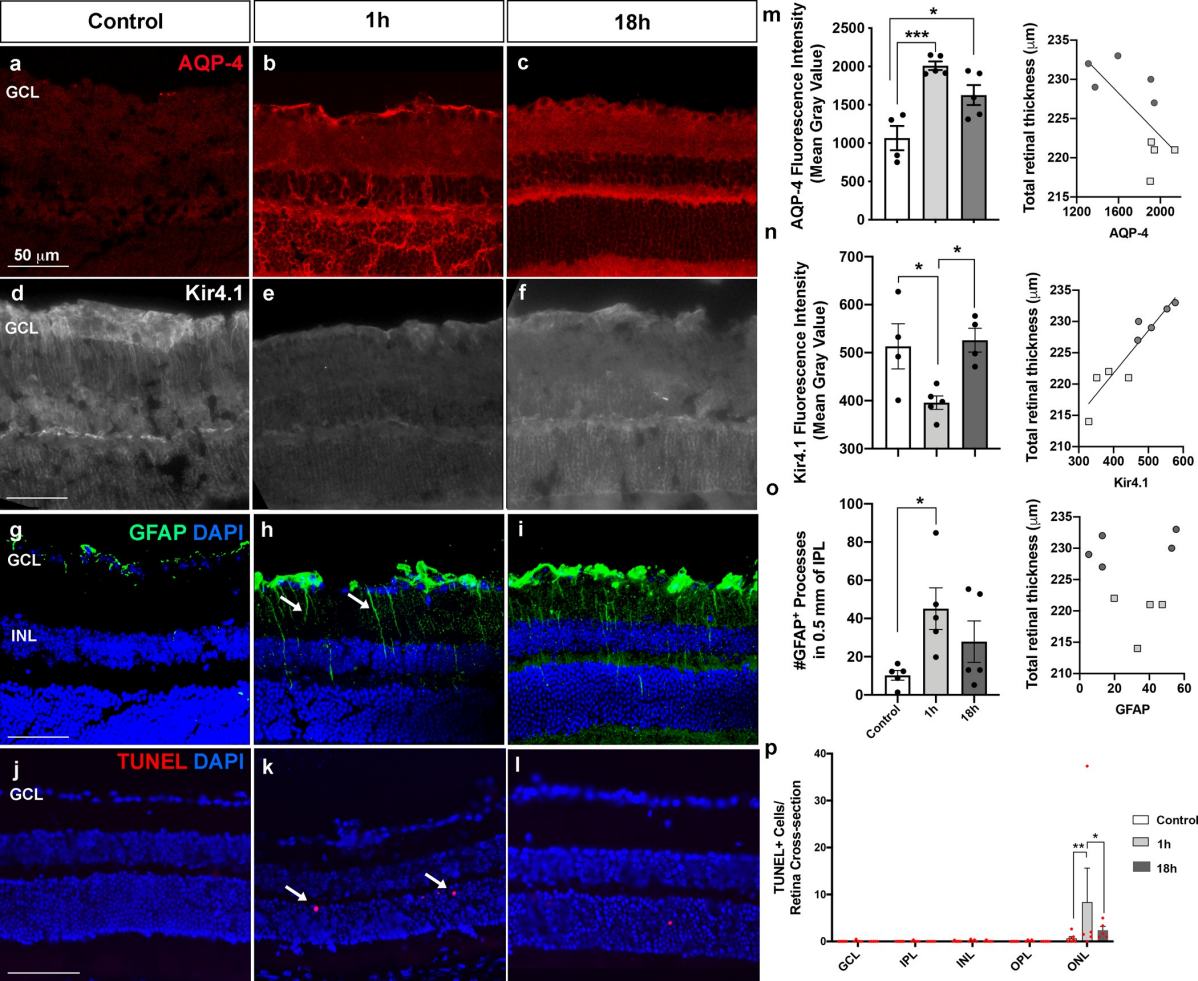
Posthypoxic effects in retinal water/ion channels, gliosis and cell death. (a) AQP-4 immunostaining of retina in control (a), 1h (b) and 18h (c) animals. (d-f) Kir4.1 immunostaining. (g-i) GFAP immunostaining (green) and DAPI nuclei labeling (blue). (j-l) TUNEL (red, arrows) and DAPI (blue). (m, n) Bar graph of quantification of AQP-4 (m) and Kir4.1 (n) expression levels in the retina and correlation with retinal thickness. (o) Bar graph of quantification of GFAP^+^ processes across the IPL and correlation with retinal thickness. (p) Bar graph of quantification of the number of TUNEL^+^ cells in each retina layer per retinal section. GCL: ganglion cell layer; INL: inner nuclear layer; IPL: inner plexiform layer; OPL: outer plexiform layer, ONL: outer nuclear layer.

We used immunohistochemistry to determine whether retinal tissue edema observed with OCT reflected alterations in cellular processes. There was significant *increase* in the expression of aquaporin-4 (AQP-4), a water channel protein expressed by retinal Müller cells and astrocytes [39, 40] at both 1h and 18h after hypoxia, compared with controls (Fig 5A–C and 5m, control: 1066 ± 158 mean gray value, n = 4 animals; 1h: 2009 ± 56, n = 5 animals each, P = 0.0005; 18h: 1627 ± 131, n = 5 animals, P = 0.018). The water flow through AQP-4 is coupled to the osmotic gradient regulated by Kir4.1, an inwardly rectifying potassium channel that prevents glial swelling after osmotic stress in the CNS [41]. Consistent with loss of glia buffering capacity [42], there was a significant *decrease* in Kir4.1 after 1h, which normalized after 18h (Fig 5D–5F and 5N, control: 471.5 ± 27.3 mean gray value, n = 4, 1h: 381.3 ± 19.7, n = 5, P = 0.04 vs. control; 18h: 527.3 ± 23.9, n = 4, P = 0.28 vs. control and P = 0.003 vs. 18h). Given that Kir4.1 is mostly expressed by retinal Müller glia [39, 40, 43], we investigated Müller cell activation following hypoxia. There was a dramatic increase in the number of GFAP^+^ Müller cell processes in the 1h group retinae (Fig 5H, arrows), which normalized in the 18h group (Fig 5G–5I and 5O, control: 10.2 ± 2.5 processes/0.5mm, n = 5, 1h: 45.1 ± 10.9, n = 5, P = 0.04, 18h: 27.9 ± 10.8, n = 5). Despite these prominent changes in glial activation and water regulation, there was little evidence of cell death. We found rare TUNEL^+^ cells in the outer retina in the 1h and 18h retinae (Fig 5J–5L and 5P) but not cell death in the inner retina (Fig 5J–5L). Overall, increased inflammatory molecules 1h after hypoxia were primarily associated with changes in glia, which are important in regulating retina homeostasis, leading to retinal tissue swelling/edema.

## Discussion

Previous studies in systemic hypoxia in humans and animals, including from our laboratory, have shown that hypoxia is associated with retinal and optic nerve structural changes consistent with edema and inflammation and that retinal and optic nerve glial changes predominate after 48h hypoxia [30], while retinal vascular changes are common after 3-week hypoxia [36]. In this study, we examined the retinal, optic nerve, and plasma changes after 1-week hypoxia–a clinically relevant time point–and found that by 18h of posthypoxic recovery, serial OCT revealed significant new thickening of the peripapillary retina consistent with posthypoxic retinal edema. This was preceded by posthypoxic systemic and CNS inflammation, with 10 significantly elevated molecules in plasma and 4 in retina, with IL-1β and VEGF increased in both. Molecular changes were most prominent immediately after hypoxia, at the 1h time point, where there was peak plasma and retinal immune molecules and significant upregulation of retinal AQP-4 and downregulation of Kir4.1 –both expressed by retinal glial cells and important for regulation of water transport. Although we do not know the origin of those immune molecules or whether some immune molecules migrated to the retina from blood, we did demonstrate that severe systemic hypoxia leads to prominent posthypoxic retinal changes. Our data support a future of imaging and molecular diagnostics in patients with CNS or systemic diseases where ophthalmic imaging and molecular profiling can help elucidate the inflammatory landscape of individual patients, which helps with disease diagnosis, monitoring, and targeting of therapeutics using a precision health-based approach [44]. Our findings on hypoxia-induced inflammation and CNS changes tracked by ocular imaging can contribute to the understanding and monitoring of cardiopulmonary conditions as well as in COVID-19-associated pneumonia and acute respiratory distress syndrome [45, 46].

We applied a targeted proteomics technique using Luminex xMAP technology to profile, for the first time, changes in 39 plasma and retinal inflammatory molecules within 24h after exposure to severe hypoxia for 1 week. This approach has been applied effectively to understand the large scale immune changes in pathogen infection [47] and has been used to study human health such as in aging [48], exercise [49] and cardiopulmonary disease [50]. In the immediate posthypoxic period, we found 10 significantly increased inflammatory proteins in the plasma including IL-6, IL-13, VEGF, and IL-1β. By profiling a large number of immune molecules using Luminex array, we identified multiple molecular changes associated with systemic hypoxia, some of which have been previously reported in humans and animals exposed to hypoxia. For example, elevated blood levels of IL-6, TNF-α and IL-1β have been reported in high-altitude-associated hypoxia [35] and in experimental hypoxia [51–58] using enzyme-linked immunosorbent assay (ELISA) to measure a small number of cytokines. In the retina, we found that 4 molecules were upregulated in systemic hypoxia: VEGF, IL-1β, IL-22, and MCP3. Retinal immune profiling for systemic hypoxia has not previously been reported prior to our study, but upregulation of immune molecules such as VEGF and IL-1 has been well-reported in retinal vascular diseases associated with hypoxia, such as proliferative diabetic retinopathy [59, 60], macular edema associated with branch retinal vein occlusion [61] and central retina vein occlusion [62–64]. IL-6, TNF-α and IL-1β upregulation and evidence of CNS inflammation have been well-reported in animal models of systemic hypoxia [65–67].

These immune proteins may be protective or detrimental. Some of these changes may be adaptive, leading to increased blood flow and tissue oxygenation [68, 69]. In our study, we found evidence of a robust inflammatory response in the post-hypoxic period after only 1 week in hypoxia. However, evidence of retinal angiogenesis such as increased retinal vessel area and branching was only found after 2 to 3 weeks of hypoxia, as described by previous studies from our group and others [36, 37]. Hypoxia-induced angiogenesis is a hallmark of ocular diseases such as diabetic retinopathy and vascular occlusion, although vessel hemorrhaging, which is common in these diseases, is not observed in whole-body hypoxia, suggesting different disease mechanisms [37]. This is consistent with an adaptive role of angiogenesis in whole-body hypoxia, however, hypoxia and the subsequent hyperoxia during the post-hypoxia recovery may lead to an exacerbated inflammatory response and negative consequences such as mitochondrial dysfunction, oxidative stress and increased levels of pro-inflammatory cytokines [54, 55].

In addition to inflammatory changes immediately after hypoxia, we found evidence of retinal glial reactivity and altered levels of AQP-4 and Kir4.1. This early glial activation in the retina is consistent with optic nerve glia vulnerability after short exposure to hypoxia [30] and with the important role of glia in spatial buffering through modulation of AQP-4 [39, 41]. Despite normalization of inflammatory proteins, there was retina edema, which was relatively delayed and observed at 18h. The role of AQP-4 and Kir4.1 in the regulation of water transport and edema formation has been well-described in retinal ischemia-reperfusion, ocular inflammation, retinal detachment and diabetes [42, 70, 71]. Consistent with increased glial reactivity after hypoxia, we found evidence of activation of retinal Müller glia [39, 72, 73], a typical response to retinal disease and injury. Müller glia increased expression of AQP-4 and consequent cell swelling may have contributed to the retinal edema observed in OCT. Although our findings support a predominant glial response to hypoxia, there was significant cell death in the outer nuclear layer, consistent with photoreceptor vulnerability to hypoxia [74, 75] and inflammation [76]. Increased AQP-4 and CNS edema have been described after hypoxic or inflammatory stimulus [35, 66, 77–79] and associated with increased VEGF and IL-1β [80, 81]. This is consistent with our findings in the plasma and retina and with a role of hypoxia in inducing first inflammatory protein and glial changes, followed by tissue edema in the CNS that can be monitored by ocular imaging.

Curiously, immediately after removal from the chamber, the OCT measurements showed significant retinal thinning. Although we do not know why, this was a very consistent finding [30] and may reflect a decrease in metabolic activity during hypoxia. As posthypoxic inflammation develops, this thinning of retina evolved to swelling, and future study to more finely delineate the hour-by-hour time course of retinal OCT changes and histologic correlation is needed. Ocular imaging studies have been done in hypoxia associated with pulmonary or cardiac disease, but they have mostly described retinal vascular changes [36] and oxygen levels [82], but not assessed retinal edema. OCT is a non-invasive technique that does not require pupil dilation, has high resolution and fast image acquisition [83, 84]. While OCT is not currently used in the Emergency Department and hospital, where hypoxia patients are, it could be useful for hypoxia assessment. For instance, routine assessment of whether hypoxia induces acute OCT changes and if these normalize over time would help predict long term CNS outcome. Our study supports natural history plasma and OCT studies in individuals at risk of systemic hypoxia. Given the importance of vascular changes in systemic hypoxia, which we have shown in patients with chronic pulmonary hypertension [36], future hypoxia studies should include OCT angiography (OCTA), which can be performed rapidly at the same time as OCT typically without pupillary dilatation. OCTA imaging can provide high-resolution vascular imaging to analyze capillary changes at different retinal layers in vivo. We have shown that using custom MATLAB script we can analyze the superficial capillary plexi around the optic nerve and macula with large vessel removal to measure 6 parameters per image, including vessel area density, vessel skeletal (or length) density, vessel complexity index (a measure of vascular tortuosity), vessel perimetric index, and flux [85].

### Limitations

Limitations of our study include the inability to perform in vivo imaging without affecting the results while the animals were inside the hypoxia chamber. We only used female young adult mice in our study due to the predominance of diseases like pulmonary hypertension in women and the desire to compare the 1-week study with our previous study of 48h hypoxia, which was done in female mice [30]. Further study will be needed to assess the changes in male or older adult mice. Although we only focused on the period less than 24h after hypoxia, the changes in the acute hypoxic period may be particularly important determinants of long-term outcome and posthypoxic neurodegeneration. For instance, retinal hypoxia and treatment in the immediate posthypoxic period have been known to be critical for visual prognosis in retinopathy of prematurity [86]. Although we did not perform functional measurements of vision in our study, this will be an important future assessment during hypoxia to determine whether systemic hypoxia model replicates the electrophysiological changes seen in humans. We anticipate that changes in the oscillatory potentials of the ERG of healthy individuals exposed to 15 min of hypobaric hypoxia [27] may be consistent with inner retinal dysfunction and with retinal edema and gliosis in our study. Using a combination of OCT and, potentially, electrophysiology after hypoxia for 2 days, 7 days, or 2–4 weeks or longer, we will be able to determine whether longer hypoxic exposure increases the risk of visual dysfunction and whether physiological adaptation occurs after certain duration of hypoxic exposure leading to relative normalization of retinal and optic nerve changes. Concomitant OCTA in addition to OCT in the same animals can help assess retinal capillary plexi in all layers of the retina in order to determine the earliest time point of hypoxia-induced retinal and optic nerve structural and vascular changes and whether angiogenesis occurs in association with evidence of inflammation and VEGF elevation.

## Conclusion

Severe systemic hypoxia leads to systemic and retina inflammation characterized by increased levels of several proteins including VEGF and IL-1β in both tissues and retinal structural changes, glial reactivity and imbalanced osmotic and water regulation. This posthypoxic response evolved into retinal edema observed using non-invasive ophthalmic imaging with OCT, supporting that more OCT studies are performed in patients at risk for systemic hypoxia to monitor CNS involvement. The significance of tissue-specific protein changes should be further investigated in disease-modelling studies, but our findings suggest that there may be consideration of treatment targeting inflammation in the posthypoxic period. This may be unwarranted in patients with self-limited retinal edema and little visual symptoms but worthy of consideration, such as what has been seen in severe SARS-CoV-2 infection [87, 88]. By performing a large inflammatory array of plasma and retina after systemic hypoxia, we can start to profile the myriad of immune molecules, how they may be connected, and which pathways are critical in posthypoxic CNS insult.

## Methods

### Animals

Animal care and experiments were carried out with approval from the Stanford University Administrative Panel on Laboratory Animal Care and all experiments were conducted in accordance with the guidelines and regulations of the approved animal use protocol. Adult wild-type C57BL/6 female mice (Charles River Laboratories, Inc., Hollister, CA, USA) were housed in cages at constant temperature, with a 12:12h light/dark cycle, with food and water *ad libitum*. Female mice were used because pulmonary hypertension, a form of systemic hypoxia, is more prevalent in females [89–91]. All efforts were made to minimize animal suffering. For procedures that required anesthesia, animals were monitored and warmed by a heat pad until they recovered.

### Hypoxia and experimental design

We induced normobaric hypoxia in adult (6–8 weeks old) C57BL/6 female mice using a hypoxia chamber where animals were acclimated over 20 min from 20.9% to 10% oxygen as described previously [92, 93]. This percentage of oxygen is consistent severe hypoxia in humans [94] and mice [95]. Mice were exposed to hypoxia for 1w and monitored daily without opening the chamber so the hypoxic exposure was unaffected. They appeared healthy, calm, and exhibited no change in behavior. After 1w hypoxia, animals were removed from the chamber, transferred to the lab, anesthesized, underwent pupillary dilation, analyzed with OCT, and then sacrificed in order to collect blood, retinae, and optic nerves (see more below). Animals in the 1h group had OCT and tissue collection as quickly as possible after removal from chamber, including OCT measurements at 20–60 min and tissue collection at 30–90 min posthypoxia. We designated this group as the 1h group because 1h was the median time of tissue collection (the last step) for each cage. Animals in the 18h group was removed from the chamber for 17h before starting OCT measurements and tissue collection. The 18h is the approximate median time for tissue collection for each cage. Normoxic control mice were kept outside the chamber in the same animal facility.

### Optical coherence tomography (OCT) and segmentation

To measure retinal structural changes over time, we performed spectral-domain optical coherence tomography (OCT) analysis using Spectralis™ HRA+OCT instrument (Heidelberg Engineering, GmbH, Heidelberg, Germany) [96–98]. Briefly, we dilated the eyes with 1% tropicamide (Alcon Laboratories, Inc., Fort Worth, TX) and 2.5% phenylephrine hydrochloride (Akorn, Inc., Lake Forest, IL) and covered the cornea with lubricating eye drops and custom-made contact lens. To measure retinal thickness, we performed a circular retinal nerve fiber layer (RNFL) scan around the optic nerve head and manually segmented a) the total retinal thickness (TRT), which included RNFL to retinal pigmented epithelium (RPE) [99], b) the ganglion cell complex (GCC), which included RNFL to inner plexiform layer (INL) [100, 101], c) the INL to RPE, which was calculated by subtracting b from a, and d) the inner and outer segments (IS/OS) of photoreceptors [99]. All segmentation was performed in a masked fashion, and every effort was made to standardize the segmentation process, which was performed by one well-trained individual and confirmed by a second investigator.

### Fresh tissue collection and plasma isolation

Animals were deeply anesthetized with ketamine and xylazine and thoracotomy was performed to expose the heart. Using a syringe coated with EDTA, the blood was drawn from the right ventricle and collected into EDTA-coated tubes (BD Microtainer® Tubes, K2 EDTA, BD 365974, BD Biosciences, San Jose, CA) and immediately placed on ice. Blood tubes were spun at 1,000–2,000xg for 10min at 4°C. Plasma was collected and stored at -80°C. Retinas were dissected and flash frozen in dry ice and then stored at -80°C. Retina lysates were prepared using RIPA buffer (ab156034, Abcam) and protease inhibitor (Mini EDTA-free protease inhibitor tablets, Roche, Basel, Switzerland).

### Immune profiling

Plasma or retinal (lysates at 0.6μg/μl) immune molecules were simultaneously measured using Luminex xMAP® Technology using microbeads (Luminex, Austin, Texas, USA) and processed by the Stanford University Human Immune Monitoring Center. We used mouse 39-plex Procarta kits (eBiosciences/Affymetrix/Thermo Fisher, Santa Clara, California, USA) and measured mean fluorescence intensity for best accuracy [102], which allowed us to compare the amount of immune molecule in each sample. All samples were run at the same time with controls, and average fluorescence intensity was calculated for each molecule and sample. For quality control, each well included 4 Chex internal control beads (Radix Biosolutions, Georgetown, Texas). Briefly, we first prepare the microbeads containing antibodies against 39 molecules (one antibody type per bead, 50–100 beads per antibody) by adding them to a 96-well plate and washing them in a Biotek ELx405 washer. Plasma or retinal samples were then added onto the plate and incubated (room temperature for 1 h then overnight at 4°C with orbital shaking at 500-600rpm). The next morning, we washed the plate with a Biotek ELx405 washer and then added biotinylated detection antibody and incubated for 75min at room temperature with shaking. The plate was washed again as above, and streptavidin-phycoerythrin (fluorescence label) was added and incubated for 30min at room temperature. Finally, we washed the plate, added reading buffer to each well, and measured fluorescence intensity using a FM3D FlexMap instrument. Luminex results are expressed as mean fluorescence intensity averaged between duplicates for each sample and normalized against Chex #4 values.

### Luminex data analysis

We performed a permutation test and selected the proteins with the smallest possible p-value, which is equivalent to having no overlap between post hypoxic groups and controls. We also performed a Mann-Whitney test using Benjamini-Hochberg multiple hypothesis correction with target false discovery rate (FDR) 0.1. Venn diagrams were generated using Venny (https://bioinfogp.cnb.csic.es/tools/venny/index.html) and edited to include relevant information.

### Tissue preparation and sectioning

Tissue preparation and sectioning was performed as previously described [30]. Animals were deeply anesthetized and perfused through the heart with ice-cold saline followed by 4% paraformaldehyde in phosphate buffered saline (PBS). Eyes and optic nerves were removed, cryoprotected with10-30% increasing sucrose gradient in PBS, and frozen in O.C.T. compound (Sakura Fineteck USA, Inc., Torrance, CA) with dry ice. Tissue blocks were sectioned using a cryostat (Leica) into 12μm thick slices and placed on frosted microscope glass slides (Fisher Scientific, Hampton, NH).

### Retina immunostaining

Retinae were immunostained with primary antibodies to detect and AQP-4 (1:50, mouse; catalog number sc-32739, Santa Cruz Biotechnology, Inc. Dallas, Texas, USA), Kir4.1 (1:200, rabbit, catalog number APC-035, Alomone Labs, Jerusalem, Israel) and glial fibrillary acidic protein (GFAP) (1:1000, rabbit; catalog number ab7260; Abcam, Cambridge, MA, USA). Secondary antibodies used were Alexa 488 goat anti-rabbit and Alexa 568 goat anti-mouse (all from Invitrogen Inc., Carlsbad, CA, USA). Slides were mounted using Vectashield with 4′,6-diamidino-2-phenylindole (DAPI) (Vector Laboratories, Burlingame, CA, USA).

### TUNEL assay

We investigated cell death in the retina as previously described [30]. We performed Terminal deoxynucleotidyl transferase (TdT) dUTP Nick-End Labeling (TUNEL) assay for the detection of apoptosis in situ. The nucleotide-labeling mix was used in combination with the TUNEL enzyme to prepare a TUNEL reaction mixture (all from Sigma-Aldrich, city, State, USA) and the assay was performed according to the manufacturer’s instructions. The number of TUNEL-positive cells was counted in 3–5 cross-sections of the retina per animal. Results are expressed as the average number of TUNEL-positive cells per section ± SEM.

### Fluorescence microscopy and image acquisition

Sections were imaged under a Nikon Eclipse TE300 microscope (Nikon Corp., Tokyo, Japan) for morphometric analysis or under a Zeiss inverted LSM 880 laser scanning confocal microscope (Carl Zeiss, Oberkochen, Germany) for representative figures.

We performed image quantification as previously described [30]. we took standard images of the retinae using a 40x (numerical aperture 0.95) objective lenses. To standardize the region of interest of the retinae quantified, we acquired 3–5 images per retina using the same objective lens, located within 1.0 mm away from the optic nerve head.

### Morphometric analysis of retina

To quantify the fluorescence intensity associated to AQP-4 or Kir4.1, slides were immunostained at the same time and imaged using the same settings. For quantification, we used ImageJ (http://rsbweb.nih.gov/ij/; provided in the public domain by the National Institutes of Health, Bethesda, MD, USA) to measure the mean gray value inside the retina. DAPI and GFAP staining were used as references to draw an outline of all retinal layers from the RNFL, including GFAP^+^ Müller cell endfeet, to the ONL. The results were expressed as mean gray value ± SEM and all morphometric analyses were performed under masked condition.

To quantify Müller glia activation, the number of GFAP+ processes per retina cross-section was counted across ~0.5 mm of the IPL in 3–5 sections per animal using a 40x lens with a field of ~0.5 mm of diameter, as previously described [72, 73]. Results are expressed ad Mean ± SEM.

### Statistics for OCT and histology

For OCT and histology data, we performed statistical analysis using Prism (GraphPad Inc.). We calculated statistical significance, which was defined as P<0.05. We used paired t-tests to compare OCT measurements at baseline and after hypoxia, One-Way ANOVA to compare 3 experimental groups for OCT measurements and immunostained retinas and two-way ANOVA to compare TUNEL+ cells across different retinal layers between 3 groups. To correct for multiple comparisons, we used Tukey’s posthoc test for all ANOVA analysis. Correlations between OCT measurements and immunostaining data were assessed by the Pearson r coefficient and P values obtained by correlation analysis. All in vivo and histological data are presented as mean ± S.E.M.

## Supporting information

S1 Fig Box plots showing the levels of proteins (mean fluorescence intensity average) in the plasma. Significantly changed proteins (P = 0.008, permutations test) are bolded and italicized. Significant proteins found using Mann-Whitney (P<0.05) are marked with a star.
(TIF)

S2 Fig Box plots showing the levels of proteins (mean fluorescence intensity average) in the retina. Significantly changed proteins (P = 0.008) are bolded and italicized.
(TIF)

S1 Table Luminex raw data for 39 immune molecules for plasma (top) and retina (bottom). All values shown are average mean fluorescence intensity (50–100 beads per molecule).
(XLSX)

## Abbreviations

AQP-4: aquaporin-4
CNS: central nervous system
GCC: ganglion cell complex
GFAP: glial fibrillary acidic protein
OCT: optical coherence tomography
ONL: outer nuclear layer
RGC: retinal ganglion cell
TRT: total retinal thickness
TUNEL: terminal deoxynucleotidyl transferase dUTP nick end labeling
IL1A: interleukin-1α
IL6: interleukin-6
LIF: leukemia inhibitory factor
RANTES: Regulated on Activation, Normal T Cell Expressed and Secreted
MCP1: monocyte chemoattractant protein-1
IFNA: interferon alpha-A
GROA: Growth-regulated alpha
IL9: interleukin-9
MCSF.CSF1: macrophage colony stimulating factor or colony-stimulating factor 1
IL13: interleukin-13
IL31: interleukin-31
IL15.IL15R: interleukin-15 and interleukin-15 receptor
MIP1A: macrophage inflammatory protein 1-α
IL3: interleukin-3
IL4: interleukin-4
GMCSF.CSF2: granulocyte-macrophage colony stimulating factor or colony-stimulating factor 2
IFNG: interferon γ
MIP1B: macrophage inflammatory protein 1-β
GCSF.CSF3: granulocyte colony-stimulating factor or colony stimulating factor 3
IL12P70: interleukin-12
MIP2: macrophage inflammatory protein 2
IL2: interleukin-2
IL27: interleukin-27
IL5: interleukin-5
IL17A: interleukin-17A
TNFA: tumor necrosis factor-alpha
IL18: interleukin-18
TGFB: transforming growth factor beta
IP10: interferon γ-induced protein 10
VEGF: vascular endothelial growth factor
IL23: interleukin-23
IL22: interleukin-22
IL1B: interleukin-1β
IL28: interleukin-28
IL10: interleukin-10
MCP3: monocyte chemotactic protein-3
