# Supplementary figures and images for: Hypoxia-induced inflammation: Profiling the first 24-hour posthypoxic plasma and central nervous system changes
Source: PLoS One. 2021 Mar 4;16(3):e0246681. doi: 10.1371/journal.pone.0246681 (PMC7932147; doi:10.1371/journal.pone.0246681)

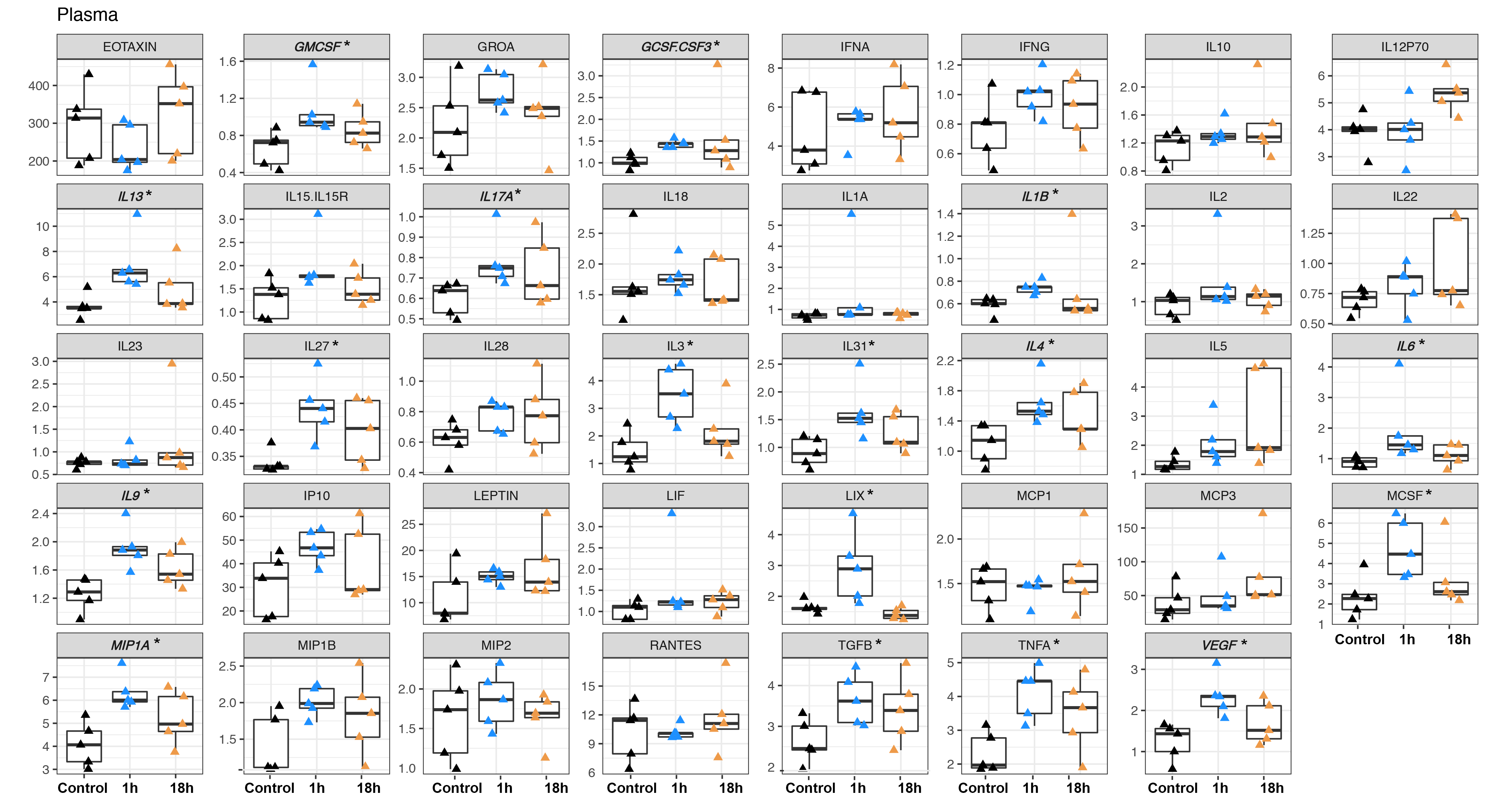

Supplement: S1 Fig — Significantly changed proteins (P = 0.008, permutations test) are bolded and italicized. Significant proteins found using Mann-Whitney (P<0.05) are marked with a star. (TIF) [file pone.0246681.s001.tif]

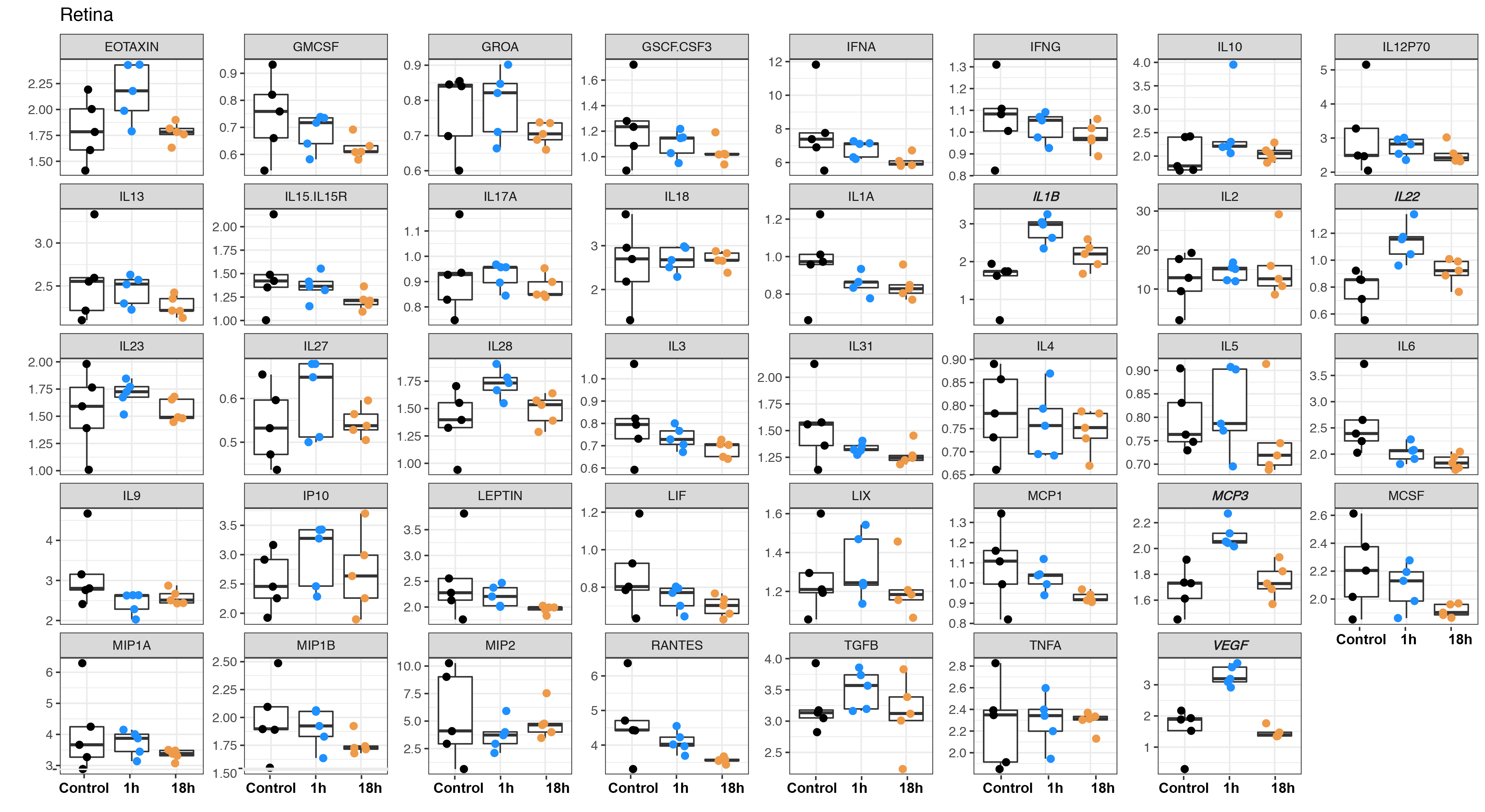

Supplement: S2 Fig — Significantly changed proteins (P = 0.008) are bolded and italicized. (TIF) [file pone.0246681.s002.tif]
